# Melioidosis: A Rare Cause of Liver Abscess

**DOI:** 10.1155/2016/5910375

**Published:** 2016-07-26

**Authors:** Peter Franz M. San Martin, Catherine S. C. Teh, Ma. Amornetta J. Casupang

**Affiliations:** ^1^Department of Surgery, National Kidney and Transplant Institute, East Avenue, Diliman, 1101 Quezon City, Philippines; ^2^Liver Center, National Kidney and Transplant Institute, East Avenue, Diliman, 1101 Quezon City, Philippines

## Abstract

*Case Presentation*. This is a case of a 44-year-old male, farmer, known to be diabetic, presenting with two-week history of vague abdominal pain associated with high grade fever. Abdominal CT scan showed localized liver abscess at segment 8 measuring 7.5 × 6.8 × 6.1 cm. Patient subsequently underwent laparoscopic ultrasound guided pigtail insertion for drainage of abscess. Culture studies showed moderate growth of* Burkholderia pseudomallei *in which the patient completed seven days of IV Meropenem. On follow-up after 12 weeks of oral Sulfamethoxazole/Trimethoprim, taken twice a day, the patient remained asymptomatic with no residual findings based on the abdominal ultrasound.* Discussion*. Diagnosis of melioidosis, a known “great masquerader,” relies heavily on culture studies. Consensus with regard to the management of liver abscess caused by* Burkholderia pseudomallei *has not yet been established due to the rarity of cases. Surgical intervention through either a percutaneous or open drainage has shown good outcomes compared to IV antibiotics alone. In Philippines, the possibility of underreporting is highly plausible. This write-up serves not only to report a rare presentation of melioidosis but also to add to the number of cases reported in the country, possibly indicative of disease emergence.

## 1. Introduction

Melioidosis has not yet been found as a public health threat in Philippines which however is highly endemic in neighboring Asian countries. For the past several years, only 2 cases have been published locally and none of these presented liver abscess.

## 2. Case Presentation

This is a case of a 44-year-old male farmer from Philippines with no history of international travel. Patient is diagnosed with diabetes mellitus, who presented with a two-week history of vague abdominal pain, localized at the right upper quadrant, and a severity score of 5 out of 10. There was no precipitating nor alleviating factors, and it was only associated with fever (*T*_max_ 40°C). The patient was admitted to a local hospital and managed as a case of typhoid fever; however, there was no improvement on IV Ampi-Sulbactam for four days. Further workup revealed a liver mass on abdominal ultrasound. The patient was then referred to our institution for further evaluation.

**Figure 1:**
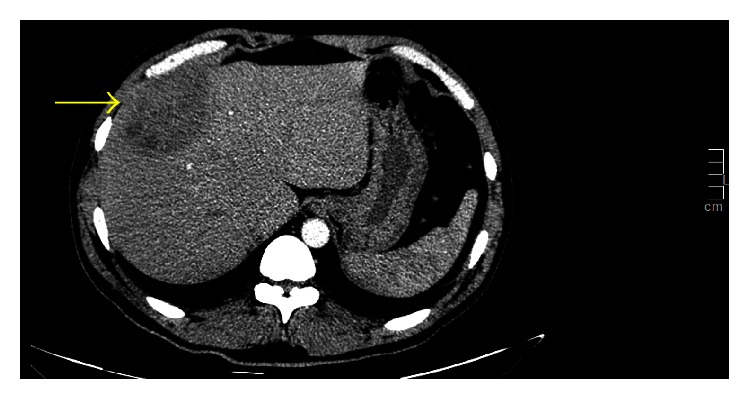
Whole abdominal triphasic CT scan showing an ill-defined, solid-cystic focus exhibiting peripheral enhancement in segment 8 of the liver measuring 7.5 × 6.8 × 6.1 cm.

The patient was received awake, febrile, tachycardic, normotensive, and with normal respiratory rate. He was anicteric and had clear breath sounds, distinct heart sounds, and a soft and nondistended abdomen with no tenderness. Laboratory tests including complete blood count, liver function tests, and metabolic profile were normal. Whole abdominal triphasic CT scan showed a liver abscess localized at segment 8 (Figure 1).

**Figure 2:**
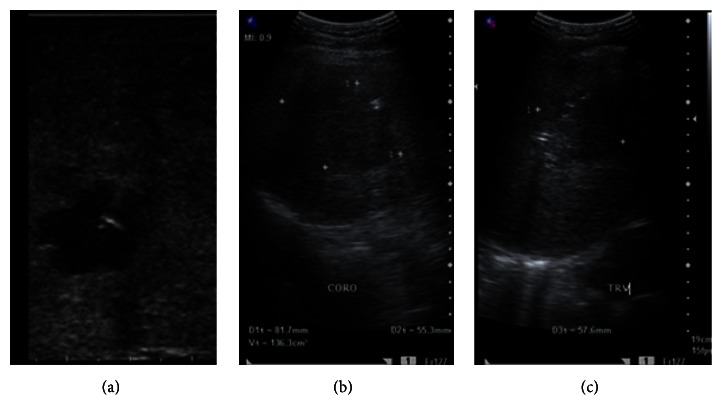
(a) Laparoscopic ultrasound showing the placement of a pigtail catheter to the liver abscess located at segment 8. ((b) and (c)) Third day's postoperative ultrasound showing decrease in the amount of abscess from 162 cc to 136 cc.

The patient was subsequently scheduled for laparoscopic guided pigtail insertion and cholecystectomy. Under laparoscopic ultrasound guidance, a 21-gauge Chiba needle was inserted into the larger cystic area and aspirated approximately 5 cc of grossly purulent material. This was followed by insertion of a 0.018-inch wire followed by coaxial dilator and a 0.038-J-tip guidewire with progressive larger dilators. Fr 10 pigtail drainage catheter was inserted and deployed within the larger cyst. Culture studies revealed moderate growth of* Burkholderia pseudomallei*. Antibiotic was then shifted accordingly: intravenous Meropenem every 8 hours for 7 days. There was note of gradual decrease in the amount of abscess drained from the pigtail as observed on serial liver ultrasound (Figure 2).

A total of 170 cc of purulent discharge was drained from the liver abscess. Pigtail was removed by the time no abscess can be drained. Residual volume was 81 cc and the patient was sent home being asymptomatic. On follow-up, 12 weeks after taking oral Sulfamethoxazole/Trimethoprim 800/160 mg twice a day, there was no recurrence of symptoms, and abdominal ultrasound showed no residual findings.

## 3. Discussion

Melioidosis is an infection caused by Gram-negative bacteria* Burkholderia pseudomallei *acquired from exposure to infected soil and water through subcutaneous inoculation, inhalation, or ingestion [1]. Risk factors include diabetes mellitus and farming [2]. Labeled as the “great masquerader,” it presents with diverse signs and symptoms which depend much on the site of infection and severity of disease. Patients often are asymptomatic or at the other end of spectrum have fulminant septic shock [2]. The most commonly reported presentations are pneumonia and skin infection [3, 4]. Isolated liver abscess however is extremely rare and its involvement is limited to disseminated cases. Liver abscess brought about by melioidosis is clinically indistinguishable to other causes of pyogenic liver abscess. It similarly presents with prolonged high grade fever however being poorly responsive to usual empiric antibiotics and occasionally may present with right upper quadrant pain and jaundice [5–8]. Abdominal CT scan findings of peripheral enhancement with “honey comb” appearance increase the suspicion especially in the presence of other affected intraabdominal organs such as the spleen [9, 10]. Definitive diagnosis remains to rely heavily on Gram staining and culture studies.

Treatment of melioidosis in general comprises intravenous Ceftazidime or Meropenem for 10 to 14 days followed by longer course of oral Trimethoprim-Sulfamethoxazole or Doxycycline for 3 to 6 months [1]. There was no consensus however on the management of liver abscess due to limited reports. Most of these cases were managed with imaging guided percutaneous drainage with concomitant recommended antibiotics. Medical management with IV antibiotics alone could be insufficient. Surgical intervention, drainage of the abscess, either percutaneously or through an open procedure, has shown good outcomes [5, 6, 8]. This report utilized the use of laparoscopic guidance for drainage of abscess due to its location on the dome of the liver which can be best drained under direct visualization via a minimally invasive approach without violating the diaphragm if done percutaneously. Liver resection as a treatment option has not yet been explored. Outcomes of nonanatomic liver resection for pyogenic liver abscess other than melioidosis have shown no recurrence rate nor need for any repeat surgical and/or radiologic intervention [11]. In addition, these cases were also found to have shorter course of IV antibiotics and hospital stay [11]. However, this option is invasive and benefit versus risk should be under the good judgment of the clinician.

There is limited knowledge about the current disease epidemiology in Philippines. Reports are scarce and most of the patients were foreign travelers who visited the country and later on their return were found to be infected with the disease [12–14]. This suggests that underreporting in the country is highly plausible and that unavailability of capacitated laboratories for diagnosis is not widely accessible. Geographical location is not explicitly cited; however, some of these mentioned cases are from the provinces of Quezon [14] and Bulacan [15] and even in Manila [13]. Agricultural areas where rice is being cultivated are known to be an endemic place for the disease. Rates were also reported in these places to be higher during monsoon and rainy season probably due to rising of water table percolating the organism [14].

As a potential lethal infection, only awareness and knowledge of the disease can equip one with a high index of suspicion. Furthermore, a mandatory reporting of diagnosed and suspected case should be accomplished to create a better picture of the disease prevalence within the country.
